# Long-term efficacy of the treat-to-close strategy for patients with atrial septal defect-pulmonary artery hypertension and characteristics of indicated populations

**DOI:** 10.1016/j.clinsp.2026.100861

**Published:** 2026-03-11

**Authors:** Jianing Fan, Yuliang Long, Jin Qi, Dawei Lin, Feng Zhang, Zhi Zhan, Dandan Chen, Wenzhi Pan, Lihua Guan, Daxin Zhou, Junbo Ge

**Affiliations:** a Department of Cardiology, Zhongshan Hospital, Fudan University, Shanghai, National Clinical Research Center for Interventional Medicine, China; b Department of Cardiology, Jinshan Hospital, Fudan University, Shanghai, China

**Keywords:** Atrial septal defect, Non-correctable, Non-repairable, Pulmonary arterial hypertension, Treat-to-Close

## Abstract

**Objective:**

“Treat-to-close” strategy in the treatment of patients with Atrial Septal Defects (ASD) combined with severe Pulmonary Hypertension (PAH). This study explored the long-term survival of patients undergoing the “treat-to-close” strategy and the characteristics of the applicable population.

**Methods:**

A total of 141 ASD-PAH patients were collected. Patients were classified as “Occlusion Group” (OG) and “Conservative Treatment Group” (CTG) depending on the suitability of ASD occlusion after drug treatment. The baseline characteristics and long-term follow-up outcomes of the two groups were recorded.

**Results:**

Patients in the OG underwent 5 (3.8) months of Pulmonary Artery-targeted Drug Therapy (PADT) before ASD occlusion. OG patients had better long-term survival (*p* = 0.043). Pulmonary Vascular Resistance (PVR): Area Under the Curve (AUC) 0.813 (0.726‒0.899, *p* < 0.001), Pulmonary Arterial Oxygen Saturation (PASO2): AUC = 0.794 (95% CI: 0.682‒0.904 *p* < 0.001), Pulmonary Artery Systolic Pressure (PASP): AUC = 0.808 (95% Confidence Interval [95% CI]: 0.733‒0.883 *p* < 0.001) and PASO2 combined with PASP: 0.918 (95% CI: 0.854‒0.979 *p* < 0.001) contribute to the screening of drug-sensitive populations.

**Conclusions:**

The treat-to-close strategy significantly improves long-term patient survival. Initial intraoperative indications can help screen the beneficiary population. Larger-scale prospective studies are needed to validate the present findings.

## Highlights

• Treat-to-close benefits atrial septal defect with pulmonary hypertension.
• Invasive hemodynamic tests identify patients who benefit from treat-to-close.
• Most drug-sensitive patients close within one year of targeted drug therapy.
• Early fall in pulmonary artery pressure with drug therapy shows a response.

## Introduction

Atrial Septal Defects (ASD) are the most common congenital heart disease in adults,^1^ with 6 %‒35 % of these patients eventually developing Pulmonary Hypertension (PAH).^2^, ^3^, ^4^, ^5^ For ASD-PAH patients, not all occlusion treatments are beneficial, especially in patients with severe PAH and irreversible remodeling of the pulmonary vascular.^6^

With the use of targeted drugs, patients with PAH experience significant improvement in clinical symptoms and survival rates.^7^, ^8^, ^9^, ^10^ Treat-and-close strategies may provide greater benefits to drug-sensitive patients.

Although the 2022 European Society of Cardiology/European Respiratory Society (ESC/ERS) guideline (section 7.5.2)^11^ considers the “treat-to-close” strategy as a therapeutic option for ASD patients combined with severe PAH, only small-scale studies have validated its efficacy.^12^, ^13^, ^14^, ^15^, ^16^ This study aimed to explore the long-term survival of patients undergoing the “treat-to-close” strategy and the characteristics of the applicable population.

## Methods

### Study patients

This study retrospectively analyzed patients with ASD complicated by PAH who were admitted to Zhongshan Hospital between December 2013 and December 2022 and underwent therapy occlusion. All patients received Pulmonary Artery-targeted Drug Therapy (PADT) for at least one month. The patients treated for ASD occlusion were classified into the Occlusion Group (OG). Patients who did not show significant improvement after at least 12-months of treatment were assigned to the Conservative Treatment Group (CTG). Patients aged < 18-years, with successful initial surgery, severe left heart failure, complex congenital heart disease, residual shunts > 3 mm after occlusion, or abandonment of treatment due to difficulties with occluder device attachment were excluded from the study cohort. The study followed the STROBE Statement and was approved by the Ethics Committee of Zhongshan Hospital, Fudan University (n° B2022–593R). As this is a retrospective study, there is no corresponding study protocol number.

### The criteria for PAH and permanent occlusion of ASD

PAH was defined as Pulmonary Artery Mean Pressure (PAMP) > 20 mmHg, Pulmonary Artery Wedge Pressure (PAWP) ≤15 mmHg, and Pulmonary Vascular Resistance (PVR) ≥ 2 Wood Units (WU) according to the latest guidelines for the management of PAH.^17^

Positive indicators for permanent occlusion, referring to the previous study,^12^ were as follows: 1) PAMP ≤ 30 mmHg within five minutes of attempted occlusion, 2) Decrease in PAMP to > 80 % immediately after occlusion compared to baseline status, 3) PVR < 5 WU; 4) Patients who did not reach a positive index at the moment of occlusion received nebulized iloprost or oxygen inhalation, with reassessment after 10 minutes.

### Clinical evaluation

Baseline patient information, including sex, age, status, underlying diseases, and echocardiography test results, was collected. For the OG, echocardiography was performed at the one-month follow-up and before the final RHC (BRHC). For CTG, echocardiographic tests were performed at the 1- and 6-month follow-ups. Patient outcomes were followed up using an electronic case system and telephone, with the endpoint of all-cause mortality.

### Statistical analysis

Continuous variables were summarized using mean ± SD. Categorical variables were summarized using frequencies and percentages. For comparison between the occlusion and conservative treatment groups, the 2-sample Student’s *t*-test for normally distributed continuous data or the Wilcoxon rank-sum test for non-normally distributed continuous data was performed. Continuous variables between different time points within a group were compared using the paired Student’s *t*-test. The Chi-Square test or Fisher’s exact test was performed for analyzing categorical variables. The Receiver Operating Characteristic (ROC) methodology was used to analyze the optimal cut-off value of variables in predicting occlusion outcomes for all enrolled patients. ROC analyses were expressed as curve plots, and the Area Under the Curve (AUC) was calculated, with the Confidence Interval (CI) and associated p-value representing the likelihood of the null hypothesis (AUC = 0.5). Cox regression analysis was conducted to identify independent predictors of a positive response. All tests were 2-tailed, and a p-value < 0.05 was considered significant. In addition, the p-values in multiple tests were adjusted using the Bonferroni method. All statistical analyses were performed using SPSS Version 25 (IBM Corp., Armonk, NY, USA).

## Results

**Fig. 1:**
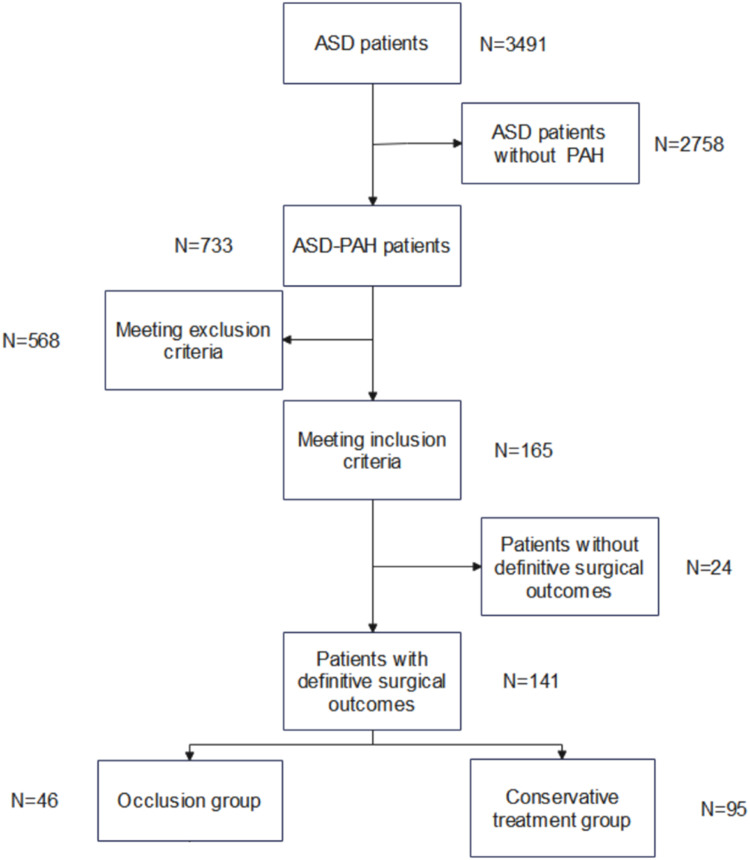
Screening process for ASD patients. ASD, Atrial Septal Defect; PAH, Pulmonary Arterial Hypertension.

A total of 3491 patients were included, of which 733 (21.0 %) had PAH. A total of 175 patients (23.8 %) underwent PADT after RHC testing. A total of 24 patients were lost to contact during follow-up. Ultimately, 141 patients aged 43.4 ± 17.2 years, of whom 34 (23.7 %) were men, were enrolled in the study. A total of 46 (32.9 %) experienced ASD occlusion after PADT (Fig. 1).

### Baseline characteristics of OG and CTG

There were no significant differences between the two groups in terms of baseline characteristics, including age, sex, and underlying diseases (Table 1).

**Table 1:** Baseline information of occlusion group and conservative treatment group.

	**OG (*n* = 46)**	**CTG (*n* = 95)**	**p-value**
**Male**	12 (25.5 %)	22 (22.9 %)	0.73
**Age (year)**	46.2 ± 19.0	43.8 ± 16.3	0.44
**Hight (mm)**	164.3 ± 6.8	162.7 ± 6.7	0.35
**Weight (kg)**	56.5 ± 10.6	57.1 ± 9.9	0.81
**BMI**	20.9 ± 3.31	21.5 ± 3.17	0.42
**Hypertension**	6 (13.0 %)	8 (8.3 %)	0.56
**AF**	8 (17.4 %)	10 (10.4 %)	0.24
**CAD**	0 (0 %)	3 (3.1 %)	0.55
**Hyperlipidemia**	0 (0 %)	2 (2.1 %)	0.99
**Hyperthyroidism**	1 (2.2 %)	3 (3.1 %)	0.99
**Diabetes**	4 (8.7 %)	3 (3.1 %)	0.31
**Stroke**	0 (0 %)	1 (1.0 %)	0.99
**Cirrhosis**	0 (0 %)	2 (2.1 %)	0.99
**HB**	132 ± 8.3	137 ± 12.1	0.152
**ALB**	26.32 ± 13.65	26.83 ± 14.11	0.863
**Creatinine**	73.11 ± 28.88	70.33 ± 17.86	0.588

AF, Atrial Fibrillation; ALB, Albumin; CAD, Coronary Heart Disease; HB, Hemoglobin; OG, Occlusion Group; CTG, Conservative Treatment Group.

### RHC test of OG and CTG

**Fig. 2:**
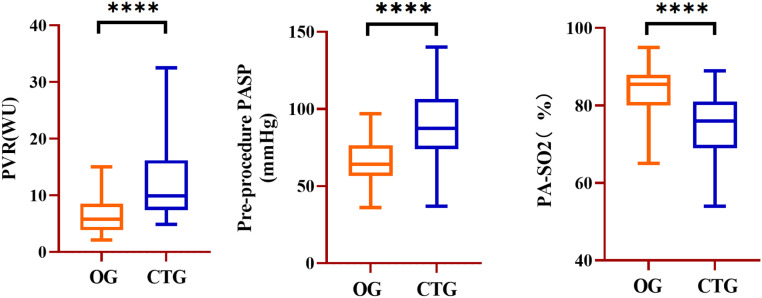
Comparison of PVR, PASP, and PA-SO2 between OG and CTG at baseline. PVR, Pulmonary Vascular Resistance, PA-SO2, Pulmonary Arterial Oxygen Saturation; OG, Occlusion Group; CTG, Conservation Treatment Group; WU, Wood Unit; **** *p* < 0.001.

Compared to the OG, CTG had higher Pulmonary Artery Systolic Pressure (PASP) (67.1 ± 14.9 vs. 89.6 ± 22.5 mmHg, *p* < 0.001), PAMP (38.7 ± 10.3 vs. 52.1 ± 14.6 mmHg, *p* < 0.001), Pulmonary Artery Diastolic Pressure (PADP) (23.7 ± 12.5 vs. 29.1 ± 12.5 mmHg, *p* = 0.012), PVR (6.47 ± 3.18 vs. 13.1 ± 8.2 WU, *p* < 0.001) Qp:Qs (2.2 ± 1.7 vs. 1.66 ± 0.90, *p* = 0.027) and significantly lower PA-SpO2 (83.0 ± 6.7 vs. 75.8 ± 8.2 %, *p* < 0.001) and SCV-SpO2 (70.4 ± 8.9 vs. 67.8 ± 9.6 %, *p* = 0.029) (Fig. 2 and Table 2).

**Table 2:** RHC information of the occlusion group and the conservative treatment group.

	**OG (*n* = 46)**	**CTG (*n* = 95)**	**p-value**
**PASP (mmHg)**	67.1 ± 14.9	89.6 ± 22.5	<0.001
**PAMP (mmHg)**	38.7 ± 10.3	52.1 ± 14.6	<0.001
**PADP (mmHg)**	20.9 ± 10.6	29.1 ± 12.5	<0.001
**LAMP (mmHg)**	6.1 ± 4.8	6.6 ± 6.2	0.66
**RAMP (mmHg)**	5.3 ± 4.4	5.9 ± 5.2	0.51
**Moderate to severe TR (%)**	58.7	71.9	0.13
**Qp:Qs**	2.21 ± 1.75	1.66 ± 0.90	0.027
**PVR (WU)**	6.47 ± 3.18	13.1 ± 8.2	<0.001
**PA-SO2 (%)**	83.0 ± 6.7	75.8 ± 8.2	<0.001
**SCV-SO2 (%)**	70.4 ± 8.9	67.8 ± 9.6	0.029
**ASD size (mm)**	23.6 ± 8.1	24.3 ± 9.9	0.71
**Occluder size (mm)**	30.6 ± 7.5	29.7 ± 9.1	0.65

PASP, Pulmonary Artery Systolic Pressure; PAMP, Pulmonary Artery Mean Pressure; PADP, Pulmonary Artery Diastolic Pressure; Qp:Qs, Preoperative pulmonary to systemic blood flow; PVR, Pulmonary Vascular Resistance; PA-SO2, Pulmonary Artery Oxygen Saturation; SCV-SO2, Superior Vena Cava Oxygen Saturation; ASD, Atrial Septal Defect.

### Postoperative medication regimens of OG and CTG

After the first RHC examination, all patients received PADT therapy, including Endothelin Receptor Antagonist (ERA), Phosphodiesterase-5 (PDE5) inhibition; Prostacyclins (PCs). Dosing regimens can be categorized based on the number of drugs into single-drug, double-drug, and triple-drug regimens. There was no significant difference between the two groups regarding the type of drugs used and the number of drugs administered (Table 3).

**Table 3:** RHC information of occlusion group and conservative treatment group.

	**OG (*n* = 46)**	**CTG (*n* = 95)**	**p-value**
**ERA**	40 (87 %)	82 (86.3 %)	0.705
**PDE-5 inhibition**	43 (93.5 %)	83 (87.3 %)	0.141
**PCs**	2 (4.3 %)	9 (9.5 %)	0.317
**Medication regimen**			0.602
**Single-drug**	9 (19.5 %)	22 (23.2 %)	
**Double-drug**	34 (73.9 %)	68 (71.6 %)	
**Triple-drug**	3 (6.5 %)	5 (5.3 %)	

ERA, Endothelin Receptor Antagonist; PDE5, Phosphodiesterase-5; PCs, Prostacyclins.

### Echocardiography test for OG and CTG

**Fig. 3:**
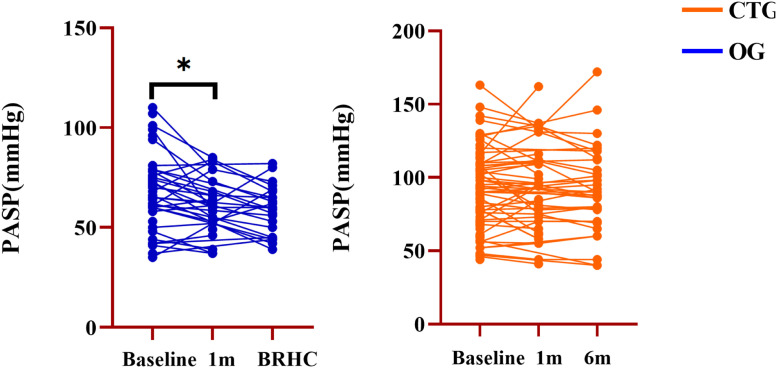
Echocardiography test follow-up in the OG and CTG. PASP, Pulmonary Artery Systolic Pressure; BRHC, Before the final Right Heart Catheterization; OG, Occlusion Group; CTG, Conservation Treatment Group; * *p* < 0.05.

Only PASP was significantly lower in the OG at one month compared to the baseline (67.7 ± 17.9 vs. 61.1 ± 15.2; *p* = 0.03) (Fig. 3 and Table 4).

**Table 4:** Echocardiography test follow-up in the two group.

**Echocardiography test follow-up of OG (*n* = 46)**					
	**Baseline**	**1-month**	**P1**	**BRHC**	**P2**
**PASP (mmHg)**	67.7 ± 17.9	60.4 ± 12.4	0.03	58.9 ± 12.4	0.24
**LVEF (%)**	65.8 ± 7.0	67.1 ± 3.6	0.67	66.6 ± 5.0	0.37
**LAD (mm)**	39.6 ± 8.6	40.2 ± 7.2	0.94	39.7 ± 6.7	0.24
**LVDD (mm)**	39.5 ± 5.2	41.9 ± 5.8	0.21	41.9 ± 4.7	0.25
**LVSD (mm)**	25.7 ± 4.6	26.3 ± 3.9	0.36	26.9 ± 3.8	0.51
**Moderate to severe TR (%)**	58.7	45.7	0.14	41.3 %	0.28
**Echocardiography test follow-up CTG (*n* = 95)**					
	**Baseline**	**3-month**	**P1**	**6-month**	**P2**
**PASP (mmHg)**	86.7 ± 26.3	87.3 ± 25.3	0.37	86.0 ± 27.9	0.60
**LVEF (%)**	67.2 ± 6.2	65.4 ± 5.5	0.48	66.7 ± 6.2	0.82
**LAD (mm)**	38.6 ± 8.8	38.3 ± 6.5	0.84	38.7 ± 8.4	0.97
**LVDD (mm)**	39.5 ± 5.6	39.6 ± 4.3	0.95	41.0 ± 5.6	0.23
**LVSD (mm)**	24.7 ± 4.7	25.2 ± 6.7	0.58	25.9 ± 4.4	0.25
**Moderate to severe TR (%)**	71.9	69.8	0.75	72.9	0.87

P1, Comparison of Pre-procedure and 1-month echocardiography test; P2, Comparison of 1-month to BRHC echocardiography test of OG, and 1-month to 6-month echocardiography test of CTG; PASP, Pulmonary Artery Systolic Pressure; LVEF, Left Ventricular Ejection Fraction; LAD, Left Atrium; LVDD, Left Ventricular Diastolic Diameter; LVSD, Left Ventricular Systolic Diameter; TR, Tricuspid Regurgitation.

### Survival of patients in OG and CTG

**Fig. 4:**
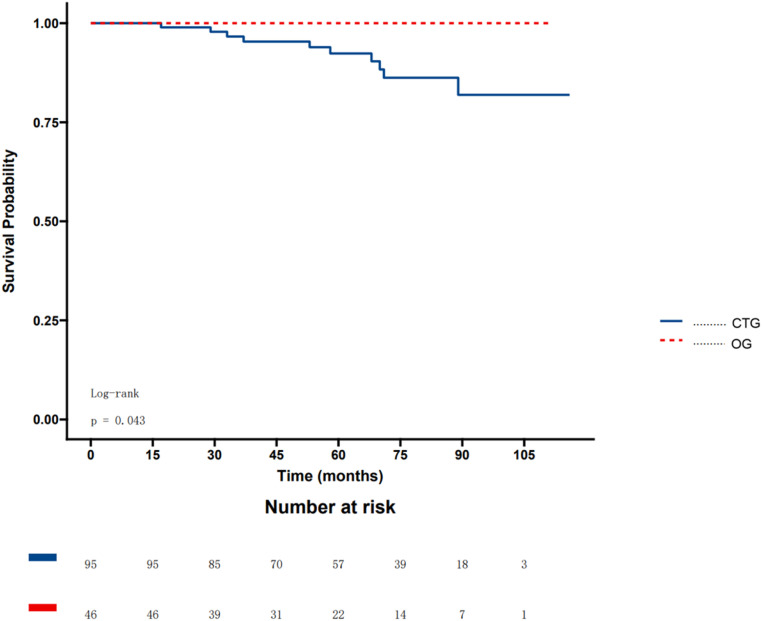
Rates of all-cause mortality in OG and CTG. OG, Occlusion Group; CTG, Conservation Treatment Group.

Patients in OG and CTG were followed up for 59.8 ± 25.9 and 64.7 ± 24.3 months (*p* = 0.18), respectively. Kaplan-Meier analysis showed better survival in OG than in CTG (*p* = 0.043) (Fig. 4).

### Cox regression analysis to explore the relationship between various parameters and final occlusion outcomes

Univariate linear regression analysis showed that PVR, PASP, Qp:Qs, and PA SO2 significantly influenced the likelihood of patients with PAH who received ASD occlusion. Further multiple linear regression analysis showed that Pulmonary Arterial Oxygen Saturation (PASO2) (OR = 1.114; 95 % CI: 1.006‒1.234, *p* = 0.038) and RHC PASP (OR = 0.959; 95 % CI: 0.924‒0.995, *p* = 0.027) were independent predictors for distinguishing between the two groups of patients (Table 5).

**Table 5:** Logistic regression analysis to explore the relationship between various parameters and final occlusion outcomes.

**Variables**	**Univariate analysis**			**Multivariate analysis**		
	**OR**	**95%CI**	**p**	**OR**	**95%CI**	**p**
Age	0.992	0.972‒1.012	0.44			
Gender	0.857	0.673‒1.187	0.78			
PVR	0.827	0.752‒0.910	<0.001	0.949	0.838‒1.075	0.41
RHC-PASP	0.969	0.955‒0.984	<0.001	0.959	0.924‒0.995	0.027
ASD size	1.026	0.979‒1.075	0.28			
Qp:Qs	1.276	1.030‒1.581	0.025			
PA-SO2 %	1.139	1.061‒1.223	<0.001	1.114	1.006‒1.234	0.038
CSV-SO2 %	1.042	0.901‒1.14	0.84			

PASP, Pulmonary Artery Systolic Pressure; PAMP, Pulmonary Artery Mean Pressure; PADP, Pulmonary Artery Diastolic Pressure; Qp:Qs, Preoperative pulmonary to systemic blood flow; PVR, Pulmonary Vascular Resistance; PA-SO2, Pulmonary Artery Oxygen Saturation; SCV-SO2, Superior Vena Cava Oxygen Saturation; ASD, Atrial Septal Defect; RHC, Right Heart Catheterization; AC, Attempted Closure.

### ROC curve to predict the optimal PVR, PASO2, and PASP values for predicting whether patients with PAH got ASD occlusion

**Fig. 5:**
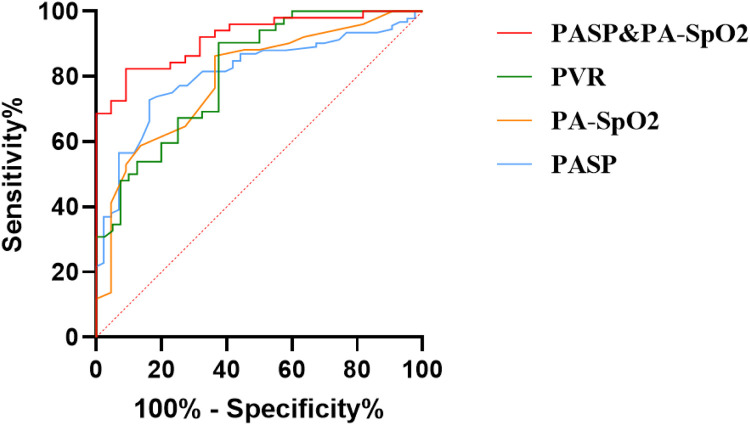
ROC curve to predict the optimal PVR, PA-SO2, and PASP values for the outcomes of closure treatment in patients. PASP, Pulmonary Artery Systolic Pressure; PVR, Pulmonary Vascular Resistance; PA-SO2, Pulmonary Arterial Oxygen Saturation.

The mean AUC of PVR for patient classification was 0.813 (95 % CI: 0.726‒0.899, *p* < 0.001), with an optimal cut-off value of 6.06 WU. At this point, the sensitivity was 90.8 %, and the specificity was 64.5 %. The AUC of PASO2 for patient classification was 0.794 (95 % CI: 0.682‒0.904, *p* < 0.001), with an optimal cut-off value of 83.5 %. At this cut-off, the sensitivity was 66.7 %, and the specificity was 86.3 %. The AUC of PASP for patient classification was 0.808 (95 % CI: 0.733‒0.883 *p* < 0.001), with an optimal cut-off value of 78.5 mmHg, yielding a sensitivity of 68.1 % and a specificity of 85.9 %. The combined AUC of PASO2 and PASP for predicting patients in the OG was 0.918 (95 % CI: 0.854‒0.979, *p* < 0.001) (Fig. 5).

## Discussion

In this study, the authors investigated the long-term survival and characteristics of patients with ASD and PAH undergoing the “treat-to-close” strategy to confirm its safety and efficacy. In this study, the authors established the following:1) Patients in OG had better long-term survival. 2) Patients in the OG showed a significant decrease in PASP in the short term after PADT. 3) Patients who were drug-sensitive completed the therapeutic regimen in less than a year. 4) PVR, PASO2, and PASP can help predict the population suitable for treat-to-close strategies.

Several clinical studies in recent years have confirmed improvements in the hemodynamic status of patients with PAH-ASD using the treat-to-close strategy.^7^^,^^18^, ^19^, ^20^ However, despite a trend, significant survival improvements were not observed. In a Multicenter Registry study conducted in North America, the average PVR was < 6.4 WU for the OG.^7^ The previous studies found that PAMP < 30 mmHg^13^ is a favorable predictor of a positive occlusion criterion that is associated with a better prognosis.^21^ In this study, the recommended PVR for ASD occlusion is in line with the previous study. All patients’ PAMP was < 30 mmHg in the OG after drug therapy. In a large study population, better survival outcomes were observed in the OG group for the first time.

The timing of pre-interventional dosing was explored for the first time, with half of the studied patients reaching the indication for occlusion after 5-months and the majority of patients in OG completing the treat-to-close strategy within a year. In contrast to the CTG, the OG showed a significant decrease in ultrasound PASP 1-month after PADT. However, the imaging characteristics of the two populations did not change during subsequent follow-up. In the OG, this could be the result of a massive cardiac shunt that could not be reversed with medication.

In addition, Cox regression analysis showed that PVR, PA-SO2, and PASP were significantly correlated with patient screening. PVR is one of the most widely used reference indices for occlusion therapy in patients with ASD-PAH.^13^ PVR responds to resistance to blood flow by the vessel wall as well as to vascular resistance due to elastic diastole and contraction. Compared to patients with lower PVR, those with higher PVR experience more severe vascular remodeling and may be less sensitive to drugs.^22^ Oxygen saturation of the superior vena cava blood and inferior vena cava blood mixes in the right ventricle and gradually becomes pulmonary arterial blood, which reflects the systemic tissue oxygen supply and is also an indicator of the sum of the cardiac output, arterial blood oxygen content, and oxygen consumption. Studies have shown that PA-SpO2 is a strong prognostic marker in patients with PAH.^22^^,^^23^ PASP is the most intuitive and powerful predictor of long-term survival in patients with PAH. A high PASP is also a predictor of persistent PAH after ASD occlusion, illustrating the difficult-to-correct lung tissue remodeling profile in such patients.^3^, ^24^, ^25^

RHC remains the gold standard for reassessing surgical indications for surgery in patients with ASD-PAH receiving pharmacological therapy. Frequent premature catheterization may result in unnecessary physical impairment and financial costs. Caution should be exercised when deciding whether surgery should be performed for CTG. These conclusions may help provide a more standardized and secure treat-to-close process.

### Limitation

This study has some limitations. First, this was a single-center retrospective analysis with a proportion of missed visits, which may have caused bias in the analysis. Second, considering the availability of eligible subjects in the ASD population and the rarity of adverse events (such as death), larger-scale prospective studies are required to enable high-level evidence subgroup analyses. Third, patients treated with open-heart surgery were excluded, and the predictors derived may have been relatively conservative in terms of the timing of occlusion. Fourth, considering that patients with Eisenmenger syndrome are currently contraindicated for defect closure,^12^ their inclusion may introduce substantial heterogeneity and confounding; nonetheless, their management and prognosis remain clinically important.

## Conclusion

In conclusion, the “treat-to-close” strategy significantly improved long-term patient survival. The initial invasive test indications help to screen the beneficiary population. Larger prospective studies are needed to validate the present findings.

## Ethical approval

This study was conducted in accordance with the STROBE Statement and was approved by the Ethics Committee of Zhongshan Hospital, Fudan University (n° B2022–593R). Informed consent was obtained from all patients before enrollment in this study.

## Authors’ contributions

Ge Jun-bo, Zhou Da-xin, Pan Wen-zhi, and Guan Li-hua, Conceptualization, Methodology and Writing-Review & Editing, Long Yu-liang, Fan Jia-ning, and Chen Dan-dan, Data Curation and Writing-Original Draft. Jin Qi, and Lin Da-wei, Formal analysis and Writing-Original Draft. Zhan Zhi, Zhang Feng, and Lin Da-wei, Visualization and Writing-Original Draft.

## Funding

Shanghai Clinical Research Center for Interventional Medicine (n° 19MC1910300); 10.13039/501100001809National Natural Science Foundation of China (n° 82,200,456)

## Data availability

The data that support the findings of this study are not publicly available due to their containing information that could compromise the privacy of research participants but are available from the corresponding author (E-mail: daxin_zhou@163.com or guan.lihua@zs-hospital.sh.cn) upon reasonable request.

## Declaration of competing interest

The authors declare no conflicts of interest.
